# Hypoxia-Related lncRNA Prognostic Model of Ovarian Cancer Based on Big Data Analysis

**DOI:** 10.1155/2023/6037121

**Published:** 2023-04-07

**Authors:** Yu Zhang, Jing Zhang, Fei Wang, Le Wang

**Affiliations:** ^1^Department of Gynecology, Shaanxi Provincial Peoples Hospital, Xi'an 710068, China; ^2^Department of Neurology, Shaanxi Provincial Peoples Hospital, Xi'an 710068, China

## Abstract

**Background:**

Hypoxia is regarded as a key factor in promoting the occurrence and development of ovarian cancer. In ovarian cancer, hypoxia promotes cell proliferation, epithelial to mesenchymal transformation, invasion, and metastasis. Long non-coding RNAs (lncRNAs) are extensively involved in the regulation of many cellular mechanisms, i.e., gene expression, cell growth, and cell cycle.

**Materials and Methods:**

In our study, a hypoxia-related lncRNA prediction model was established by applying LASSO-penalized Cox regression analysis in public databases. Patients with ovarian cancer were divided into two groups based on the median risk score. The survival rate was analyzed in the Cancer Genome Atlas (TCGA) and International Cancer Genome Consortium (ICGC) datasets, and the mechanisms were investigated.

**Results:**

Through the prognostic analysis of DElncRNAs (differentially expressed long non-coding RNAs), a total of 5 lncRNAs were found to be closely associated with OS (overall survival) in ovarian cancer patients. It was evaluated through Kaplan–Meier analysis that low-risk patients can live longer than high-risk patients (TCGA: *p* = 1.302*e* − 04; ICGC: 1.501*e* − 03). The distribution of risk scores and OS status revealed that higher risk score will lead to lower OS. It was evaluated that low-risk group had higher immune score (*p* = 0.0064) and lower stromal score (*p* = 0.00023).

**Conclusion:**

It was concluded that a hypoxia-related lncRNA model can be used to predict the prognosis of ovarian cancer. Our designed model is more accurate in terms of age, grade, and stage when predicting the overall survival of the patients of ovarian cancer.

## 1. Introduction

Ovarian cancer is a type of malignant tumor that cannot be easily detected in the early stage and has a poor prognosis. There are many risk factors associated with its occurrence and development, i.e., family history of ovarian or breast cancer, obesity (BMI of 30 and above), genetic mutations, delayed menopause, fertility treatments, polycystic ovary syndrome, and smoking. The mortality rate of this type of tumor is ranked fifth among other female malignant tumors [1]. Due to the concealment of the incidence of ovarian cancer, more than half of the patients lost the opportunity for early diagnosis, which seriously affects its prognosis [2]. Surgery plus chemotherapy is a classic treatment for ovarian cancer. First-line maintenance therapy, including bevacizumab or PARP inhibitors, can prolong progression-free survival (PFS), which is different from OS [3, 4]. Therefore, it is necessary to explore more treatments to prolong the lifespan of ovarian cancer patients. At present, immunotherapy is the new major therapeutic tool of ovarian cancer. However, the effect of single immunotherapy for ovarian cancer is not obvious [5, 6].

When malignant tumors increase in their size, tumors gradually form a hypoxia environment, due to which the cancer cells undergo some adaptive changes, such as proliferation and angiogenesis [7]. The direct reaction of molecules to reverse hypoxia is to stabilize the HIFs. Otherwise, HIF can enhance cell viability and increase angiogenesis and cell invasion. HIF can help in the survival of cancer cells that can undergo apoptosis [8, 9]. Hypoxia can also alter the immune microenvironment of malignant tumors [10]. In ovarian cancer, hypoxia promotes cell proliferation [11], epithelial to mesenchymal transformation, invasion, and metastasis [12]. The aforementioned phenomenon may lead to higher mortality of patients [13].

lncRNAs consist of more than 200 nucleotides and can interact with various kinds of biomolecules such as DNA, RNA, and proteins, which have attracted increasing attention. lncRNAs are extensively involved in the regulation of many important cellular processes, such as gene expression, dosage compensation, and regulation of the cell growth and cell cycle [14]. lncRNAs may play their role in the nucleus as well as in the cytoplasm. The lncRNAs can act as modulators to affect the protein-coding gene expression by regulating the transcriptional and post-transcriptional processes. As there is difference in expression of IncRNAs in various tissues, some of them have been identified for their implication in the pathogenesis of the diseases, such as tumor, neurological, cardiovascular, and orthopedic disease. Recent evidence suggests that lncRNAs precipitated the malignant phenotype of cancer through genomic or transcriptional changes. In addition to these changes, changing the immune environment may also promote the malignant phenotype [15, 16]. However, a study on hypoxia-related lncRNA in ovarian cancer is still need of the hour. Therefore, a hypoxia-related lncRNA model was established in our study. This model can be applied to pre-calculate the prognosis of ovarian cancer. More importantly, the immune status can be predicted by this model, which can act as a guiding tool for better clinical treatment.

## 2. Materials and Methods

### 2.1. Data Acquisition

TCGA database (TCGA-OV, 379 samples) (https://tcga-data.nci.nih.gov/tcga/) and the ICGC portal (OV-AU, 81 samples) (https://dcc.icgc.org) were used to extract the RNA sequence data. At the same time, the corresponding clinical features were also downloaded. One of the inclusion criteria of the study was that the patients must survive more than 30 days. The gene expression profiles of normal ovarian tissue as a controlled study were downloaded from the GTEx database (88 samples).

### 2.2. Obtention of Genes and lncRNAs That Are Related to Hypoxia

We downloaded 200 genes related to hypoxia (Table S1 of GSEA). In TCGA database, using Pearson correlation (|*R*| > 0.4, *p* < 0.001), 1330 lncRNAs that have a relationship with hypoxia were selected. Then, the difference analysis was performed by a limma R package. To screen the hypoxia-related differentially expressed lncRNAs (DElncRNAs), we set the standard as (FDR) < 0.05 and |log2FC| ≥ 1.

### 2.3. Risk Scoring of Candidate Genes for Hypoxia-Related lncRNAs

To identify candidate genes for hypoxia-related lncRNAs from TCGA cohort, we analyzed OS by univariate Cox analysis. Firstly, we built the prognostic model using LASSO-penalized Cox regression analysis. The risk score was calculated using the following formula.

(1) $$\begin{array}{c} \text{RiskScore}=\sum \left(CoeCCL\text{lnc}RNACC\times \text{expressCConoCClncRNACC}\right)\text{.} \end{array}$$

Ovarian cancer patients were then categorized into two groups on the basis of median of TCGA cohort risk scores and named as high or low risk. PCA was performed with the “stats” R packages to explore the distribution of the groups. OS of the two groups was analyzed by Kaplan–Meier analysis. ROC curves with “survival ROC” R package were plotted for 1/3/5 years. Cox regression was utilized to predict the independent values. The above analyses were carried out simultaneously in TCGA and ICGC datasets. After that, the nomogram including risk, grade, stage, and age was set up by the “rms” R package. Finally, we plotted the correction curve to evaluate the difference between the predicted values and actual values.

### 2.4. GSEA

GSEA between the two groups was performed in the gene set with the parameter kegg.v7.4.symbols.gmt. To detect the significantly enriched pathways, the criterion was *p* < 0.05 and FDR < 0.05.

### 2.5. Immunity Analysis

The following methods were used to calculate the immune penetration status between TCGA project samples, including XCell, TIMER, QUANTISEQ, EPIC, CiberSort-ABS, and CiberSort. All these methods are in silico techniques that were used to integrate the advantages of gene enrichment. These are deconvolution techniques to reanalyze the data in comprehensive way [17, 18]. Meanwhile, we compared the TME scores between the two risk groups by the “ggpubr” R package.

### 2.6. Division of Clusters by Risk Model

Using the “Consensus Cluster Plus” R package, two molecular subgroups were grouped based on the prognostic model in ovarian cancer patients. Kaplan–Meier survival analysis, PCA, and tSNE were performed. In addition, analysis of immune-related indexes including immune infiltration cells and TME scores was carried out between the two molecular subgroups.

## 3. Results

### 3.1. Hypoxia-Related lncRNAs in Ovarian Cancer

**Figure 1:**
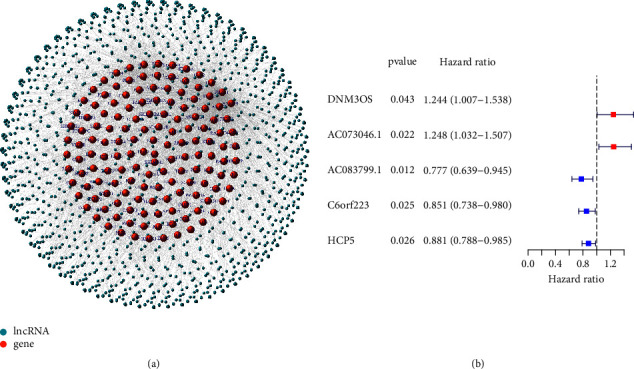
(a) The network of the hypoxia-related genes and lncRNAs. (b) DElncRNAs related to prognosis.

In ovarian cancer, we identified 1330 hypoxia-related lncRNAs. The network of the hypoxia-related genes and lncRNAs is presented in Figure 1(a). There were a total of 145 hypoxia-related DElncRNAs, of which 111 lncRNAs were downregulated, and 34 were upregulated (Table S2). Through the prognostic analysis of DElncRNAs, a total of 5 lncRNAs were found closely associated with OS of ovarian cancer patients, serving as candidate lncRNAs for modeling (Figure 1(b)).

### 3.2. Evaluation of Prognostic Role of Hypoxia-Related lncRNAs by Risk Model

**Figure 2:**
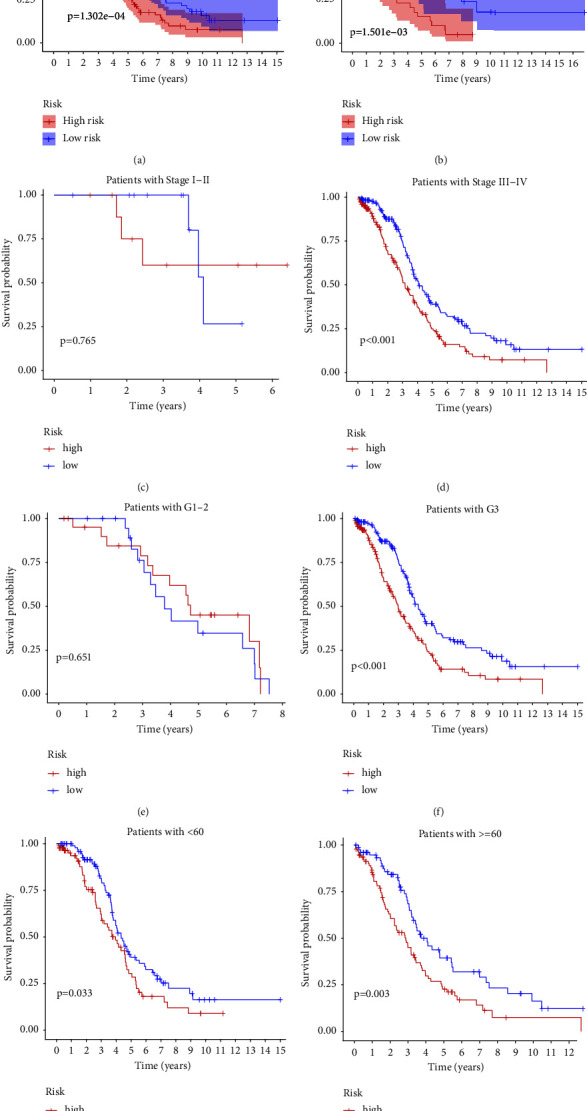
Kaplan–Meier analysis in TCGA cohort (a) and ICGC cohort (b). Kaplan–Meier analysis of the OS time in different clinical groups in TCGA cohort ((c, d) stage; (e, f) grade; (g, h) age).

We constructed a risk model including 5 lncRNAs (DNM3OS, AC073046.1, AC083799.1, C6orf223, and HCP5) in TCGA cohort. The formula we used to calculate the risk score is risk score = DNM3OS × 0.152 + AC073046.1 × 0.126-AC083799.1 × 0.184-C6orf223 × 0.097-HCP5 × 0.082. After calculations, we evaluated revisions to the risk models established in TCGA and ICGC databases. Kaplan–Meier analysis showed that low-risk patients can live longer than high-risk patients (TCGA: *p*=1.302*e* − 04; ICGC: 1.501*e* − 03) (Figures 2(a) and 2(b)). At the same time, we analyzed the OS time of patients in different clinical groups in TCGA dataset. There was no significant difference in OS time when patients were diagnosed with stage I-II or grade 1-2 (*p*=0.765, 0.651). But, significant differences were observed in OS time for the stage III-IV group (*p* < 0.001), G3 group (*p* < 0.001), under 60 years (*p*=0.033), and over 60 years (*p*=0.003) (Figures 2(c)–2(h)).

**Figure 3:**
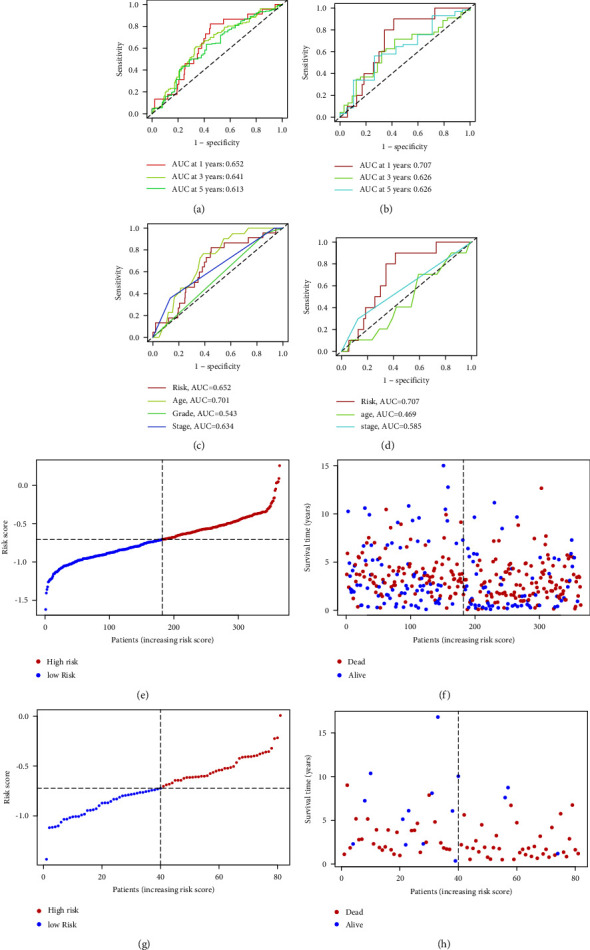
(a, b) ROC curve analysis of 1/3/5 years (a) in TCGA cohort (b) in ICGC cohort, (c, d) ROC curve analysis of risk sore and other clinicopathological features in (c) TCGA cohort, (d) ICGC cohort, (e) distribution of risk scores, (f) survival status, in the TCGA database, distribution of (g) risk scores, and (h) survival status in the ICGC database.

In ROC curve analysis, the one-year AUC in TCGA cohort was 0.652, while the 3- and 5-year AUCs were 0.641 and 0.613, respectively (Figures 3(a) and 3(c)). The value of AUC in the ICGC cohort was calculated as 0.707, 0.626, and 0.626, respectively (Figures 3(b) and 3(d)). We also found that the model was more accurate in terms of age, grade, and stage when predicting the OS. The distribution of risk scores and OS status indicated that a higher risk score will lead to a lower OS (Figures 3(e)–3(h)).

**Figure 4:**
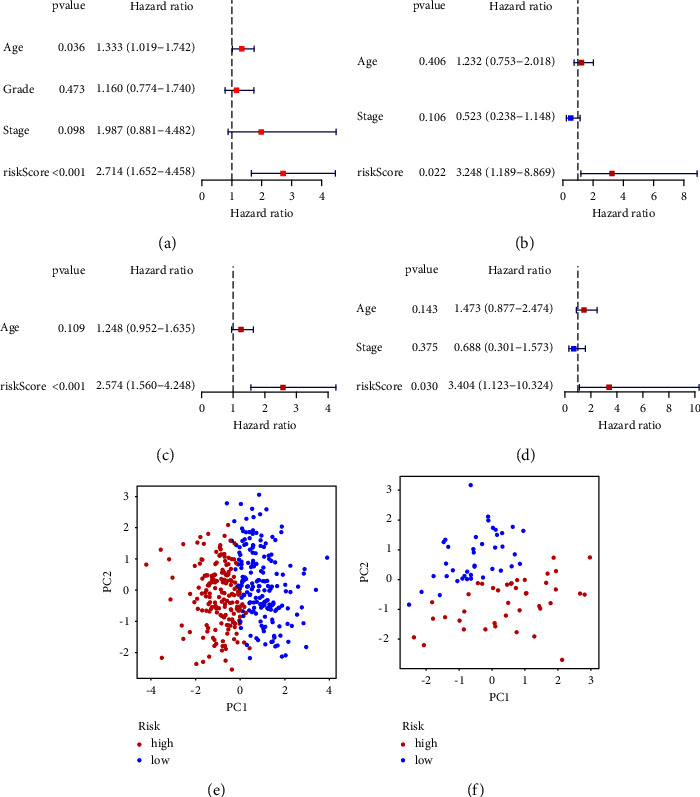
(a) Univariate Cox regression analysis in TCGA database. (b) Regression analysis in ICGC database. Multivariate Cox regression analysis in (c) TCGA database and (d) ICGC database. PCA in (e) TCGA dataset or (f) ICGC dataset.

When univariate Cox regression analysis was performed, core risk showed its association to OS. The HR value of TCGA cohort was 2.714 (95% CI = 1.652–4.458, *p* > 0.001). For the ICGC cohort, the HR value was 3.248 (95% CI = 1.189–8.869), and the *p* value was 0.022. (Figures 4(a) and 4(b)). Different from univariate analysis, multivariate analysis demonstrated an independent role of the risk score in predicting OS in the both cohorts. For TCGA cohort, the HR value was 2.574, 95% CI was 1.560 to 4.248, and the *p* value was 0.001. For the ICGC cohort, the HR value was 3.404 (95% CI = 1.123–10.324, *p*=0.030) (Figures 4(c) and 4(d)). PCA verified that the predictive model could divide ovarian cancer patients into two different groups in two datasets (Figures 4(e) and 4(f)).

**Figure 5:**
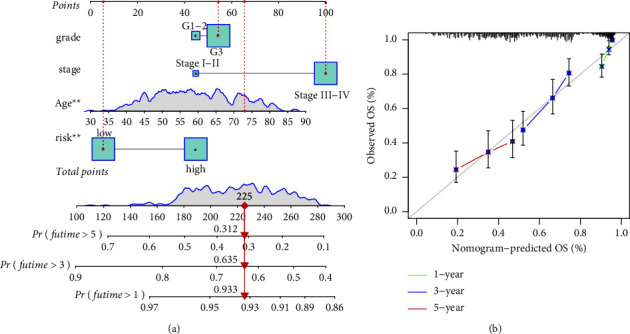
(a) Construction of nomogram including the prognostic hypoxia-related lncRNA signature and clinicopathological features. (b) The correction curve predicted the value of the nomogram in predicting prognosis.

Finally, we used other factors including risk, grade, age, and stage to predict 1-/3-/5-year OS (Figure 5(a)). Calibration chart was used to judge whether the result of the nomogram is accurate (Figure 5(b)).

### 3.3. Cancer-Related Pathways Are Enriched

**Figure 6:**
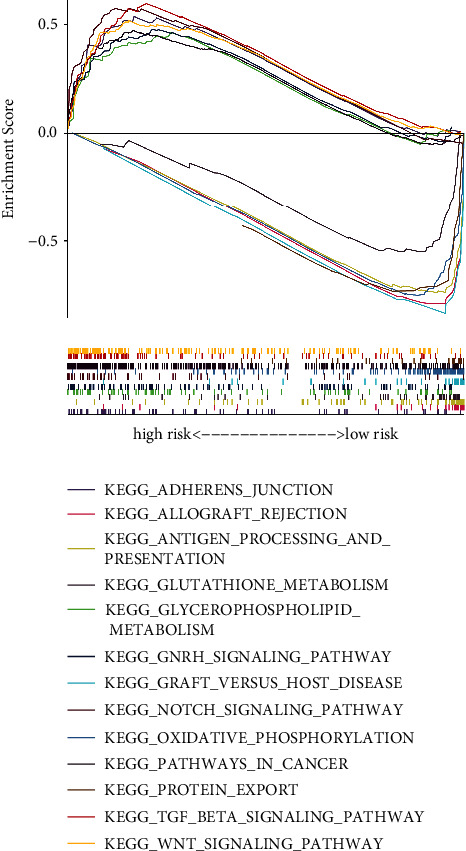
GSEA of the prognostic hypoxia-related lncRNA signature.

In the high-risk group, the pathways enriched in cancer-related pathways were adherens junction, TGF-beta, Wnt, Notch, GnRH signaling pathway, and glycerophospholipid metabolism. In the low-risk group, the pathways were enriched in oxidative phosphorylation, antigen processing, antigen presentation, graft-versus-host disease, metabolism related to glutathione, allograft rejection, and protein export (Figure 6).

### 3.4. Immune Scoring of Risks Groups Evaluated by GSEA

**Figure 7:**
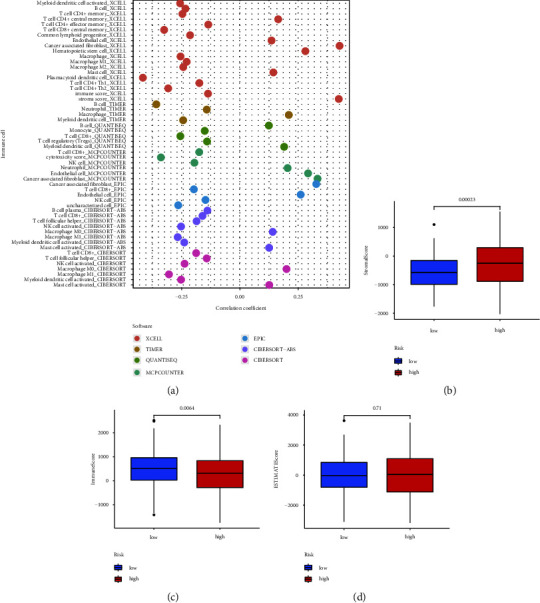
(a) The immune infiltration status of the risk model. (b, c, d) TME scores in different risk groups.

We further analyzed the immune status of the two groups with different risks in TCGA database. More M1 macrophages, myeloid dendritic, activated NK, and CD8 + T cells were detected in the low-risk group, while fewer cancer-associated fibroblasts (CAFs) and neutrophils were detected in the low-risk group (Figure 7(a), Supplementary 1). The low-risk group had a higher immune score (*p* = 0.0064) and a lower stromal score (*p* = 0.00023). However, the ESTIMATE score did not show significant differences between the two risk groups (Figures 7(b)–7(d)).

### 3.5. Analyses Related to Molecular Subtype

**Figure 8:**
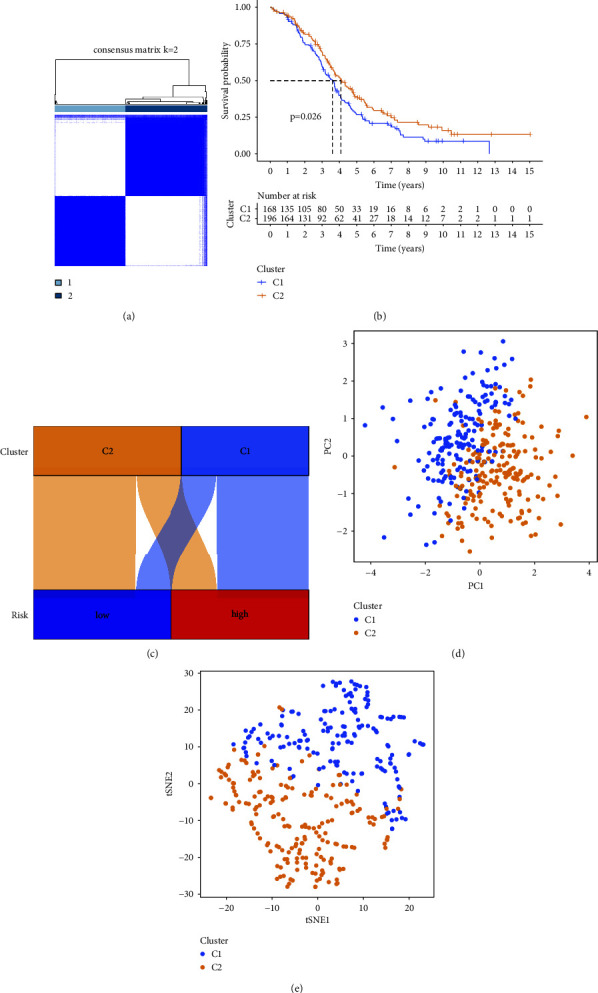
(a) Molecular subgroups according to according to the prognostic model. (b) Kaplan–Meier survival analysis of the two clusters. (c) The relationship between clusters and risk groups. PCA (d) and tSNE2 (e) of the two clusters.

Based on this hypoxia-related lncRNA model, we redivided ovarian cancer patients into two clusters (Figure 8(a)). The OS of cluster1 patients was shorter than cluster2 patients (Figure 8(b)). Most of the patients of cluster2 were in the low-risk group, while most patients of cluster1 were in the high-risk group (Figure 8(c)). PCA and tSNE2 clearly showed that patients can be grouped as two completely various subgroups (Figures 8(d) and 8(e)).

**Figure 9:**
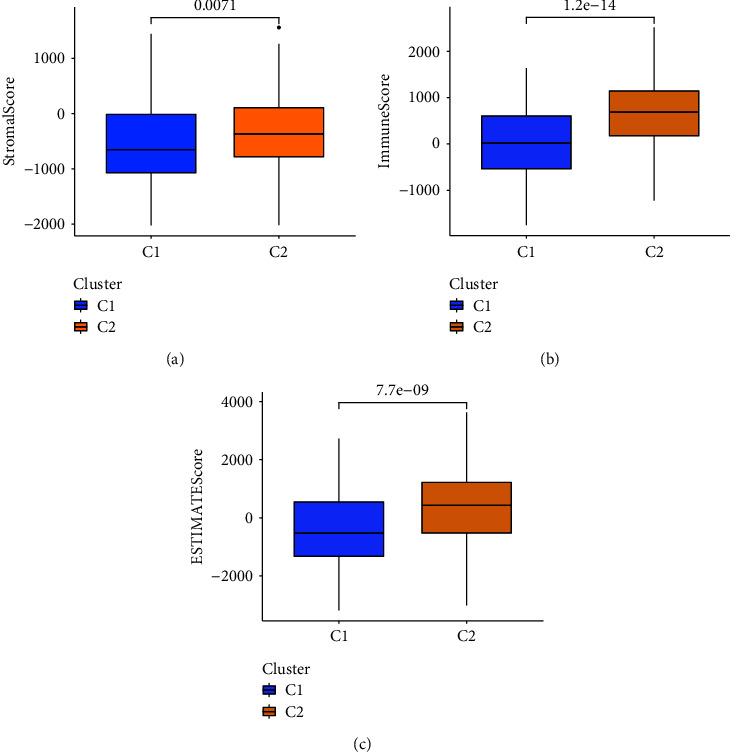
TME scores of the two clusters. (a) Stromal score of C1 and C2. (b) Immune score of C1 and C2. (c) ESTIMATE score of both clusters.

Cluster1 showed lower stromal score (*p*=0.0071) (Figure 9(a)), lower immune score (*p*=1.2*e* − 14) (Figure 9(b)), and lower ESTIMATE score (*p*=7.7*e* − 09) (Figure 9(c)).

**Figure 10:**
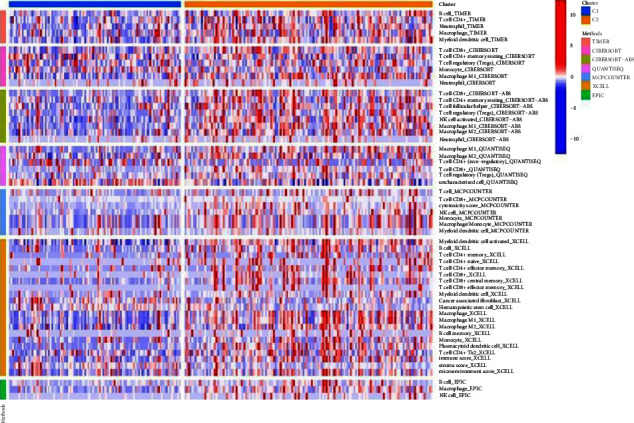
The heatmap of immune infiltration of the two clusters.

The heatmap of immune cells was drawn by using the following methods, including TIMER, CIBERSORT, CIBERSORT-ABS, QUANTISEQ, MCPCOUNTER, XCELL, and EPIC. All the graphs are presented in Figure 10.

## 4. Discussion

Ovarian cancer is a malignant disease that cannot be cured completely. Surgery, chemotherapy, and targeted therapy are the most commonly used methods for its treatment nowadays, but the prognosis remains poor [19, 20]. Many research studies have evaluated that hypoxia-related lncRNAs are involved in the progression of various cancers. Hypoxic regions are commonly found in solid tumors, and the appearance of these regions often harms the progression of tumors and triggers tumor immunosuppression and may affect the therapeutic response. However, the underlying mechanism is not fully understood. In our study, hypoxia-related lncRNA was selected as the standard to subgroup patients with various risks. Patients in different groups have different prognoses and different immune statuses. This model can help the clinicians to classify and individualize the treatment of ovarian cancer patients and inspire researchers to gain insight into the important role of hypoxia-related lncRNAs in ovarian cancer.

Hypoxia can change the repair mechanism of DNA [21], promote tumorigenesis [22] and metastasis [23, 24], and lead to the development of cancer stem cells [25, 26], which are resistant to chemotherapy and radiotherapy [27, 28]. Therefore, the relationship between hypoxia and cancer needs further study, including hypoxia-related coding genes and non-coding genes. However, it is reported that DNA damage is not induced by hypoxia; instead this hypoxia leads to some genomic instabilities [21]. The modeling of hypoxia-related lncRNA to stratify patients with malignant tumors has been a concern by some scholars. For example, related research has been carried out on hepatocellular cancer and renal cell cancer [29, 30]. In our study, a prognostic model consisting of five lncRNA was constructed, including DNM3OS, AC073046.1, AC083799.1, C6orf223, and HCP5. DNM3OS was overexpressed in ovarian cancer and facilitated ovarian cancer progression, and its high expression might lead to a poor prognosis [31]; the conclusion is similar to our research. At the same time, the role of DNM3OS in other malignant tumors has been confirmed. Tumor-associated mesenchymal stem cells can target DNM3OS, leading to the progression of hepatocellular cancer [32]. In retinoblastoma, the DNM3OS-miR-134-5p-SMAD6 axis can promote cell proliferation, migration, and the EMT process [33]. DNM3OS can also promote tumor progression in gastric cancer [34] and oral cancer [35]. HCP5 has been studied in several kinds of tumors, including ovarian [36], esophageal [37], gastric [38], and colorectal cancer [39]. It has been demonstrated that HCP5 can target miR-525-5p/PRC1 signaling pathway and can target the Wnt/beta-catenin pathway [36]. Other types of lncRNAs are presented for the first time, through our study.

In the high-risk group, enriched Wnt, Notch, TGF-beta, and tumor-related pathways were found. It has been regarded as one of the leading factors of high mortality in the high-risk group. Wnt/beta-catenin pathway played an important role in ovarian cancer cells' carcinogenesis, stemness, and resistance ability against chemotherapy [40]. The hyperactivation of the Wnt signaling pathway mediated some drug resistance in ovarian cancer, such as olaparib [41]. The synergistic effect of Wnt and Notch signaling pathway can promote the proliferation of cancer cells and further increase the migration of cancer cells [42]. Studies have shown that the Notch pathway is closely related to angiogenesis and chemotherapy resistance of ovarian cancer [43, 44]. The Notch signal pathway was regarded as the characteristic of enriched ovarian cancer stem cells induced by hypoxia [45]. TGF*β* pathway also facilitates epithelial-mesenchymal transition (EMT) of epithelial ovarian cancer (EOC) [46].

Hypoxia can promote the development of malignant tumors, including ovarian cancer. At the same time, ascites can also induce hypoxia [47]. One reason is that the function of anti-tumor immune cells is inhibited in a hypoxia environment [10]. Therefore, the immune environment of the two groups was compared. More M1 macrophages, myeloid dendritic cells, activated NK cells, and CD8 + T cells were detected in the low-risk group, while less cancer-associated fibroblasts (CAFs) and neutrophils were detected in the low-risk group. M1 macrophages have an anti-tumor effect, while M2 macrophages can promote tumor [48]. M2 macrophages were also closely related to the progression of cancer cells [49]. We found higher M1/M2 values in the low-risk group, which did not differ from previous findings, and patients with higher M1/M2 values lived longer [50]. Dendritic cells can activate T cells such as CD4+ and CD8+ cells to fight tumors by presenting an antigen [51, 52]. Other studies have shown that NK cells had abilities that could lead to ovarian cancer cell death, and they often co-infiltrate with CD8 + T cells [53]. CD8 + T cells are quite important in anti-tumor immunity, and further, we can predict patients' OS [54]. In ovarian cancer, CAF may lead to deterioration and drug resistance of the ovarian cancer [55]. Regarding the former research, ovarian tumors are generally regarded as cold tumors, which pose a challenge to the treatment. The immune activity of patients in the low-risk group tends to be “hot” and may be sensitive to immunotherapy, which provides new opportunities for patients.

lncRNAs play many roles in cancer diagnosis and treatment, but the most important role of these RNAs is to control gene expression and regulate many important biological processes in the body, such as proliferation, genome stability, apoptosis, pyrolysis, autophagy, and immunity, and dysregulation of these functions contributes to the progression of many tumors. We can conclude that the model is verified in the external dataset; the model can be further stratified through analysis of patients with different clinicopathological characteristics, which can provide more accurate guidance for clinical treatment. The limitation of study is that there is no experimental verification of lncRNA in the model. The reason is that it is impossible to accumulate enough fresh tissue specimens for survival analysis in a short period.

## 5. Conclusion

It was concluded the hypoxic microenvironment is closely related to the occurrence and development of ovarian cancer. Our established hypoxia-related lncRNA model can be applied to pre-calculate the prognosis of ovarian cancer. In addition, the immune status can be predicted using this model. Our result indicates that the hypoxia-related lncRNA model can serve as a guiding tool for better clinical treatment of ovarian cancer.

## Data Availability

TCGA database can be found at https://tcga-data.nci.nih.gov/tcga/, and the ICGC database can be found at https://dcc.icgc.org.

## Supplementary Materials

Supplementary Materials Table S1: 200 hypoxia-related genes were downloaded from Gene Set Enrichment Analysis (hallmark-hypoxia). Table S2: 145 hypoxia-related DElncRNAs (differentially expressed lncRNAs). Supplementary 1 (a, b): correlation between immune infiltrating cells and risk score.
