# Supplementary material for: Hypoxia-Related lncRNA Prognostic Model of Ovarian Cancer Based on Big Data Analysis
Source: J Oncol. 2023 Apr 7;2023:6037121. doi: 10.1155/2023/6037121 (PMC10104744; doi:10.1155/2023/6037121)

| Table s2. 145 hypoxia-related DElncRNAs(differential expressed lncRNAs) | | | | | | |
| --- | --- | --- | --- | --- | --- | --- |
| **ID** | **gene** | **conMean** | **treatMean** | **logFC** | **pValue** | **fdr** |
| DIO3OS | DIO3OS | 6.428285 | 0.329954 | -6.09833 | 2.05E-48 | 4.59E-47 |
| MIR202HG | MIR202HG | 5.357003 | 0.583813 | -4.77319 | 4.59E-47 | 3.14E-46 |
| STAG3L5P-PVRIG2P-PILRB | STAG3L5P-PVRIG2P-PILRB | 5.30679 | 0.841422 | -4.46537 | 2.05E-48 | 4.59E-47 |
| GUSBP11 | GUSBP11 | 4.217245 | 0.177137 | -4.04011 | 2.05E-48 | 4.59E-47 |
| MIR503HG | MIR503HG | 5.168397 | 1.196249 | -3.97215 | 1.07E-46 | 6.57E-46 |
| NR2F2-AS1 | NR2F2-AS1 | 3.877141 | 0.159567 | -3.71757 | 2.08E-48 | 4.59E-47 |
| LINC01001 | LINC01001 | 4.029657 | 0.37464 | -3.65502 | 2.05E-48 | 4.59E-47 |
| ADAMTS9-AS1 | ADAMTS9-AS1 | 3.886844 | 0.248995 | -3.63785 | 2.25E-48 | 4.59E-47 |
| PCBP1-AS1 | PCBP1-AS1 | 4.404513 | 1.034012 | -3.3705 | 2.05E-48 | 4.59E-47 |
| LINC00893 | LINC00893 | 4.082798 | 0.948846 | -3.13395 | 4.33E-48 | 5.31E-47 |
| KLF3-AS1 | KLF3-AS1 | 3.858156 | 0.747385 | -3.11077 | 3.67E-48 | 4.89E-47 |
| CARMN | CARMN | 3.141677 | 0.152754 | -2.98892 | 2.05E-48 | 4.59E-47 |
| AL157392.3 | AL157392.3 | 3.463068 | 0.653099 | -2.80997 | 2.56E-48 | 4.59E-47 |
| LINC01197 | LINC01197 | 2.992999 | 0.19865 | -2.79435 | 3.22E-48 | 4.72E-47 |
| SH3BP5-AS1 | SH3BP5-AS1 | 3.975683 | 1.276112 | -2.69957 | 2.91E-48 | 4.62E-47 |
| MEG9 | MEG9 | 2.890974 | 0.199179 | -2.69179 | 1.23E-47 | 1.06E-46 |
| PSMA3-AS1 | PSMA3-AS1 | 4.890409 | 2.207492 | -2.68292 | 2.05E-48 | 4.59E-47 |
| LINC01089 | LINC01089 | 4.562171 | 1.910297 | -2.65187 | 4.71E-47 | 3.20E-46 |
| AC002398.2 | AC002398.2 | 2.789986 | 0.153447 | -2.63654 | 2.84E-48 | 4.62E-47 |
| AC009955.4 | AC009955.4 | 3.175807 | 0.588054 | -2.58775 | 1.89E-46 | 1.09E-45 |
| ADAMTS9-AS2 | ADAMTS9-AS2 | 2.630839 | 0.128228 | -2.50261 | 2.16E-48 | 4.59E-47 |
| LINC00894 | LINC00894 | 3.140657 | 0.679657 | -2.461 | 6.61E-48 | 6.81E-47 |
| AL513314.2 | AL513314.2 | 2.715053 | 0.298772 | -2.41628 | 3.44E-48 | 4.78E-47 |
| FAM198B-AS1 | FAM198B-AS1 | 2.550252 | 0.190065 | -2.36019 | 2.16E-48 | 4.59E-47 |
| CCDC18-AS1 | CCDC18-AS1 | 4.126909 | 1.812934 | -2.31397 | 3.93E-46 | 2.10E-45 |
| ACTA2-AS1 | ACTA2-AS1 | 2.931008 | 0.620535 | -2.31047 | 6.36E-44 | 2.47E-43 |
| GAS5 | GAS5 | 8.479937 | 6.174074 | -2.30586 | 1.91E-45 | 9.03E-45 |
| AL117190.1 | AL117190.1 | 2.369759 | 0.102801 | -2.26696 | 9.17E-48 | 8.52E-47 |
| VLDLR-AS1 | VLDLR-AS1 | 2.683833 | 0.425802 | -2.25803 | 1.17E-46 | 7.07E-46 |
| AL162586.1 | AL162586.1 | 3.777896 | 1.55734 | -2.22056 | 1.36E-43 | 5.14E-43 |
| AC009120.2 | AC009120.2 | 3.347595 | 1.147587 | -2.20001 | 3.35E-48 | 4.76E-47 |
| AP000892.3 | AP000892.3 | 2.785626 | 0.652992 | -2.13263 | 3.66E-44 | 1.46E-43 |
| LINC02614 | LINC02614 | 2.664713 | 0.534148 | -2.13057 | 2.49E-48 | 4.59E-47 |
| AC108058.1 | AC108058.1 | 2.392425 | 0.271424 | -2.121 | 4.40E-46 | 2.33E-45 |
| TARID | TARID | 2.325255 | 0.246424 | -2.07883 | 3.22E-47 | 2.32E-46 |
| PTOV1-AS2 | PTOV1-AS2 | 3.99611 | 1.921241 | -2.07487 | 2.63E-47 | 1.95E-46 |
| SNHG22 | SNHG22 | 2.516017 | 0.54207 | -1.97395 | 2.23E-47 | 1.70E-46 |
| SEMA6A-AS1 | SEMA6A-AS1 | 2.167534 | 0.198026 | -1.96951 | 2.05E-48 | 4.59E-47 |
| AC074212.1 | AC074212.1 | 3.06891 | 1.130748 | -1.93816 | 1.35E-44 | 5.67E-44 |
| ZFHX2-AS1 | ZFHX2-AS1 | 2.241148 | 0.33225 | -1.9089 | 2.05E-48 | 4.59E-47 |
| AL135999.1 | AL135999.1 | 2.928332 | 1.019486 | -1.90885 | 1.79E-47 | 1.43E-46 |
| AC004466.1 | AC004466.1 | 2.256805 | 0.367604 | -1.8892 | 2.08E-48 | 4.59E-47 |
| AL035425.1 | AL035425.1 | 2.04525 | 0.192327 | -1.85292 | 1.39E-45 | 6.72E-45 |
| LINC00342 | LINC00342 | 3.388686 | 1.54023 | -1.84846 | 5.02E-45 | 2.22E-44 |
| DNM3OS | DNM3OS | 2.72119 | 0.887297 | -1.83389 | 3.87E-43 | 1.41E-42 |
| LINC01128 | LINC01128 | 2.614908 | 0.831907 | -1.783 | 2.11E-48 | 4.59E-47 |
| AL355987.4 | AL355987.4 | 2.070055 | 0.31764 | -1.75241 | 1.12E-47 | 9.85E-47 |
| AL359715.1 | AL359715.1 | 2.20318 | 0.464222 | -1.73896 | 5.18E-48 | 5.87E-47 |
| LINC00924 | LINC00924 | 1.933432 | 0.194791 | -1.73864 | 1.50E-47 | 1.24E-46 |
| AC025165.1 | AC025165.1 | 2.020296 | 0.293807 | -1.72649 | 7.71E-48 | 7.50E-47 |
| FAM13A-AS1 | FAM13A-AS1 | 1.928336 | 0.22168 | -1.70666 | 2.05E-48 | 4.59E-47 |
| ERVK13-1 | ERVK13-1 | 2.582971 | 0.914167 | -1.6688 | 1.10E-47 | 9.76E-47 |
| TRAF3IP2-AS1 | TRAF3IP2-AS1 | 2.454459 | 0.821405 | -1.63305 | 5.89E-48 | 6.35E-47 |
| AL021368.2 | AL021368.2 | 1.941117 | 0.344438 | -1.59668 | 2.11E-48 | 4.59E-47 |
| AL513327.1 | AL513327.1 | 2.137918 | 0.555983 | -1.58194 | 1.01E-47 | 9.10E-47 |
| AL031714.1 | AL031714.1 | 2.399501 | 0.820992 | -1.57851 | 3.06E-47 | 2.22E-46 |
| AC010636.1 | AC010636.1 | 2.133408 | 0.577609 | -1.5558 | 1.36E-32 | 2.94E-32 |
| LINC01018 | LINC01018 | 1.980367 | 0.458054 | -1.52231 | 2.71E-41 | 8.60E-41 |
| AC087500.2 | AC087500.2 | 1.939931 | 0.470179 | -1.46975 | 8.10E-48 | 7.81E-47 |
| LINC01341 | LINC01341 | 1.993334 | 0.537062 | -1.45627 | 1.26E-45 | 6.13E-45 |
| AP001107.4 | AP001107.4 | 2.235464 | 0.779869 | -1.4556 | 7.17E-43 | 2.55E-42 |
| AC109587.1 | AC109587.1 | 1.839456 | 0.394179 | -1.44528 | 2.08E-48 | 4.59E-47 |
| ZFAS1 | ZFAS1 | 7.265939 | 5.824706 | -1.44123 | 9.94E-33 | 2.15E-32 |
| HCG18 | HCG18 | 3.190386 | 1.753288 | -1.4371 | 2.14E-47 | 1.65E-46 |
| AC093110.1 | AC093110.1 | 1.956595 | 0.533388 | -1.42321 | 2.11E-47 | 1.63E-46 |
| AC005519.1 | AC005519.1 | 2.288638 | 0.870035 | -1.4186 | 4.22E-45 | 1.89E-44 |
| AL049840.3 | AL049840.3 | 2.230104 | 0.827233 | -1.40287 | 6.05E-48 | 6.45E-47 |
| AC027288.3 | AC027288.3 | 1.86979 | 0.48739 | -1.3824 | 1.23E-33 | 2.74E-33 |
| AC037198.1 | AC037198.1 | 1.84414 | 0.465473 | -1.37867 | 6.71E-38 | 1.78E-37 |
| AC008969.1 | AC008969.1 | 2.2184 | 0.848532 | -1.36987 | 9.34E-47 | 5.82E-46 |
| AC108010.1 | AC108010.1 | 2.669194 | 1.302349 | -1.36685 | 4.75E-42 | 1.59E-41 |
| AC114730.3 | AC114730.3 | 1.910229 | 0.543778 | -1.36645 | 2.70E-43 | 9.97E-43 |
| AC069281.2 | AC069281.2 | 3.001376 | 1.650896 | -1.35048 | 1.07E-38 | 2.93E-38 |
| LINC02519 | LINC02519 | 1.672952 | 0.33478 | -1.33817 | 8.85E-44 | 3.40E-43 |
| MSC-AS1 | MSC-AS1 | 1.81007 | 0.48218 | -1.32789 | 1.11E-44 | 4.70E-44 |
| AC011498.6 | AC011498.6 | 1.701594 | 0.38478 | -1.31681 | 3.21E-47 | 2.32E-46 |
| AC009133.1 | AC009133.1 | 2.501895 | 1.185764 | -1.31613 | 5.05E-45 | 2.23E-44 |
| GABPB1-AS1 | GABPB1-AS1 | 2.714661 | 1.399484 | -1.31518 | 1.34E-38 | 3.67E-38 |
| AL390208.1 | AL390208.1 | 1.696487 | 0.382842 | -1.31364 | 1.65E-46 | 9.67E-46 |
| AL450384.2 | AL450384.2 | 2.140538 | 0.832063 | -1.30847 | 6.93E-44 | 2.69E-43 |
| RAMP2-AS1 | RAMP2-AS1 | 1.60003 | 0.297533 | -1.3025 | 5.84E-44 | 2.28E-43 |
| C1RL-AS1 | C1RL-AS1 | 2.223467 | 0.932869 | -1.2906 | 6.60E-42 | 2.18E-41 |
| AL022328.1 | AL022328.1 | 2.153182 | 0.86559 | -1.28759 | 1.37E-44 | 5.74E-44 |
| N4BP2L2-IT2 | N4BP2L2-IT2 | 1.604486 | 0.32098 | -1.28351 | 3.06E-48 | 4.68E-47 |
| AC136475.1 | AC136475.1 | 1.889649 | 0.624489 | -1.26516 | 1.57E-41 | 5.06E-41 |
| AL049840.2 | AL049840.2 | 2.052413 | 0.804789 | -1.24762 | 7.27E-46 | 3.69E-45 |
| AC080013.1 | AC080013.1 | 1.675534 | 0.429889 | -1.24564 | 9.19E-44 | 3.53E-43 |
| DTX2P1-UPK3BP1-PMS2P11 | DTX2P1-UPK3BP1-PMS2P11 | 1.779111 | 0.54549 | -1.23362 | 4.44E-48 | 5.38E-47 |
| AC004492.1 | AC004492.1 | 1.673831 | 0.450353 | -1.22348 | 2.12E-46 | 1.21E-45 |
| AC084125.2 | AC084125.2 | 1.879627 | 0.660045 | -1.21958 | 1.93E-43 | 7.21E-43 |
| AC090510.2 | AC090510.2 | 1.51839 | 0.308961 | -1.20943 | 1.29E-47 | 1.10E-46 |
| FTX | FTX | 1.827224 | 0.620615 | -1.20661 | 6.70E-48 | 6.86E-47 |
| AC095057.3 | AC095057.3 | 1.770813 | 0.590732 | -1.18008 | 3.39E-41 | 1.07E-40 |
| AC002553.2 | AC002553.2 | 1.838072 | 0.660266 | -1.17781 | 2.49E-43 | 9.22E-43 |
| AC037198.2 | AC037198.2 | 1.499182 | 0.323652 | -1.17553 | 3.66E-36 | 8.97E-36 |
| MCM3AP-AS1 | MCM3AP-AS1 | 1.953671 | 0.810697 | -1.14297 | 3.99E-47 | 2.78E-46 |
| AC021078.1 | AC021078.1 | 2.435536 | 1.297484 | -1.13805 | 5.13E-40 | 1.50E-39 |
| LINC00926 | LINC00926 | 1.440323 | 0.305327 | -1.135 | 4.83E-47 | 3.27E-46 |
| AF117829.1 | AF117829.1 | 2.047891 | 0.942726 | -1.10516 | 2.89E-46 | 1.59E-45 |
| MIR22HG | MIR22HG | 3.100922 | 1.997146 | -1.10378 | 1.23E-09 | 1.56E-09 |
| Z83843.1 | Z83843.1 | 2.128189 | 1.025101 | -1.10309 | 1.50E-42 | 5.18E-42 |
| AC005104.1 | AC005104.1 | 1.882009 | 0.794468 | -1.08754 | 5.84E-43 | 2.09E-42 |
| AC008735.2 | AC008735.2 | 3.547134 | 2.460788 | -1.08635 | 7.15E-27 | 1.31E-26 |
| SPAG5-AS1 | SPAG5-AS1 | 1.440555 | 0.385018 | -1.05554 | 3.10E-47 | 2.24E-46 |
| AC087500.1 | AC087500.1 | 1.879714 | 0.828181 | -1.05153 | 1.15E-44 | 4.87E-44 |
| RAD51-AS1 | RAD51-AS1 | 3.080425 | 2.030038 | -1.05039 | 4.35E-32 | 9.18E-32 |
| AL022328.2 | AL022328.2 | 2.132681 | 1.088516 | -1.04416 | 1.62E-40 | 4.91E-40 |
| AC073869.1 | AC073869.1 | 3.909101 | 2.880013 | -1.02909 | 1.80E-31 | 3.72E-31 |
| AC005253.1 | AC005253.1 | 1.864727 | 0.846487 | -1.01824 | 2.21E-44 | 9.09E-44 |
| ZNF436-AS1 | ZNF436-AS1 | 2.465247 | 1.447125 | -1.01812 | 8.22E-33 | 1.78E-32 |
| Z97989.1 | Z97989.1 | 1.613268 | 0.595983 | -1.01729 | 7.48E-42 | 2.46E-41 |
| AL031985.3 | AL031985.3 | 0.403531 | 1.435956 | 1.032425 | 4.17E-45 | 1.86E-44 |
| HOXB-AS3 | HOXB-AS3 | 0.143791 | 1.192687 | 1.048896 | 2.28E-25 | 4.02E-25 |
| AC026471.4 | AC026471.4 | 1.136188 | 2.188185 | 1.051997 | 6.34E-36 | 1.54E-35 |
| AC139887.1 | AC139887.1 | 0.386816 | 1.44236 | 1.055544 | 4.40E-36 | 1.08E-35 |
| AL513008.1 | AL513008.1 | 0.023903 | 1.079874 | 1.055972 | 3.45E-43 | 1.26E-42 |
| AC073046.1 | AC073046.1 | 0.285455 | 1.379715 | 1.09426 | 1.34E-38 | 3.68E-38 |
| AL355803.1 | AL355803.1 | 0.111224 | 1.238111 | 1.126887 | 5.97E-39 | 1.66E-38 |
| AC006001.2 | AC006001.2 | 0.661939 | 1.834436 | 1.172497 | 1.21E-42 | 4.21E-42 |
| MIRLET7A1HG | MIRLET7A1HG | 0.390113 | 1.570279 | 1.180166 | 8.04E-36 | 1.94E-35 |
| AC020913.3 | AC020913.3 | 0 | 1.218028 | 1.218028 | 1.03E-45 | 5.08E-45 |
| LINC01614 | LINC01614 | 0.197747 | 1.462596 | 1.26485 | 2.45E-15 | 3.51E-15 |
| AC138207.2 | AC138207.2 | 0.512175 | 1.808392 | 1.296217 | 2.00E-42 | 6.86E-42 |
| AL589765.4 | AL589765.4 | 0.035496 | 1.377204 | 1.341708 | 4.60E-48 | 5.51E-47 |
| AC021683.1 | AC021683.1 | 0.170979 | 1.537927 | 1.366948 | 2.02E-35 | 4.80E-35 |
| AC138207.5 | AC138207.5 | 0.154695 | 1.527414 | 1.372719 | 1.94E-46 | 1.12E-45 |
| LINC01063 | LINC01063 | 0.142612 | 1.548584 | 1.405972 | 1.20E-45 | 5.83E-45 |
| HM13-IT1 | HM13-IT1 | 0.50403 | 1.976133 | 1.472103 | 4.18E-46 | 2.22E-45 |
| AL080317.1 | AL080317.1 | 1.028693 | 2.530992 | 1.5023 | 1.23E-41 | 3.99E-41 |
| AC087289.1 | AC087289.1 | 2.591486 | 4.140276 | 1.54879 | 2.54E-33 | 5.60E-33 |
| C6orf223 | C6orf223 | 0.037139 | 1.634498 | 1.597358 | 5.84E-48 | 6.31E-47 |
| NCK1-DT | NCK1-DT | 0.878572 | 2.477834 | 1.599262 | 5.32E-48 | 5.96E-47 |
| AL691482.3 | AL691482.3 | 0.12305 | 1.787069 | 1.66402 | 3.97E-44 | 1.58E-43 |
| TRPM2-AS | TRPM2-AS | 0.115223 | 1.839826 | 1.724603 | 3.29E-44 | 1.32E-43 |
| AC083799.1 | AC083799.1 | 1.523376 | 3.343547 | 1.820171 | 9.83E-47 | 6.07E-46 |
| AC004233.3 | AC004233.3 | 0.06466 | 2.011554 | 1.946894 | 3.52E-48 | 4.82E-47 |
| HCP5 | HCP5 | 1.453162 | 3.447623 | 1.994461 | 4.46E-37 | 1.13E-36 |
| KRT7-AS | KRT7-AS | 0.090115 | 2.167631 | 2.077517 | 7.53E-48 | 7.41E-47 |
| AL354836.1 | AL354836.1 | 1.145345 | 3.439699 | 2.294354 | 2.60E-46 | 1.45E-45 |
| AC009237.14 | AC009237.14 | 0.91844 | 3.217358 | 2.298918 | 3.10E-48 | 4.68E-47 |
| AL445524.1 | AL445524.1 | 1.15481 | 3.471746 | 2.316936 | 3.52E-47 | 2.50E-46 |
| AL390719.2 | AL390719.2 | 0.115662 | 2.491392 | 2.37573 | 1.32E-46 | 7.88E-46 |
| AC092718.4 | AC092718.4 | 0.584187 | 3.159288 | 2.5751 | 2.05E-48 | 4.59E-47 |
| AC099850.3 | AC099850.3 | 0.171959 | 2.828069 | 2.65611 | 1.87E-48 | 4.59E-47 |
| SPINT1-AS1 | SPINT1-AS1 | 0.526977 | 3.735509 | 3.208532 | 3.98E-46 | 2.13E-45 |

Supplementary Figure 1a


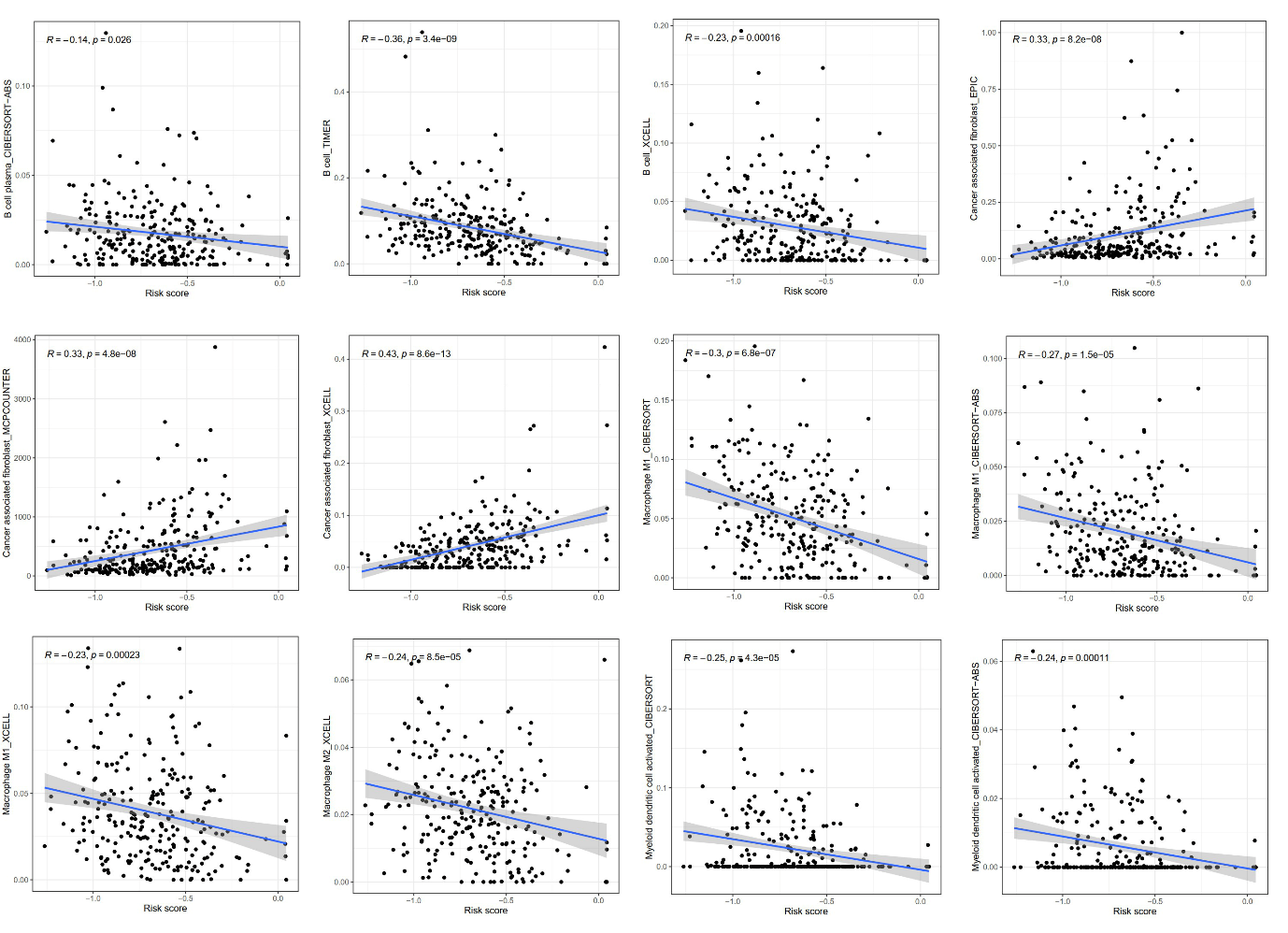


Supplementary Figure 1b


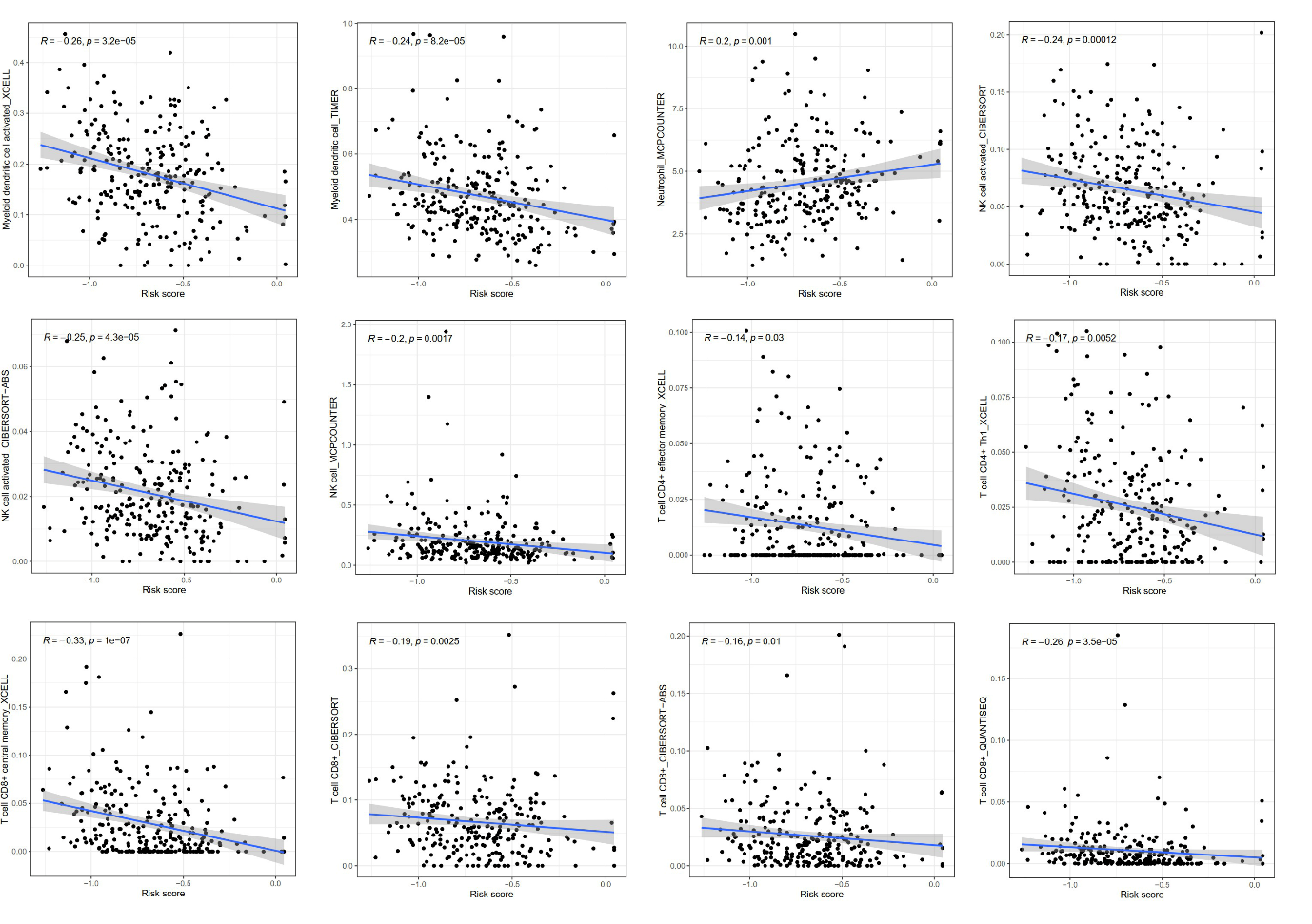

Supplement: Supplementary Materials — Table S1: 200 hypoxia-related genes were downloaded from Gene Set Enrichment Analysis (hallmark-hypoxia). Table S2: 145 hypoxia-related DElncRNAs (differentially expressed lncRNAs). Supplementary 1 (a, b): correlation between immune infiltrating cells and risk score. [file 6037121.f1.zip › supplmentary (1).docx]
