# Supplementary material for: Hypoxia-Related lncRNA Prognostic Model of Ovarian Cancer Based on Big Data Analysis
Source: J Oncol. 2023 Apr 7;2023:6037121. doi: 10.1155/2023/6037121 (PMC10104744; doi:10.1155/2023/6037121)
Supplement: Supplementary Materials — Table S1: 200 hypoxia-related genes were downloaded from Gene Set Enrichment Analysis (hallmark-hypoxia). Table S2: 145 hypoxia-related DElncRNAs (differentially expressed lncRNAs). Supplementary 1 (a, b): correlation between immune infiltrating cells and risk score. [file 6037121.f1.zip › Table s1.docx]

Table S1. The 200 hypoxia-related genes which were downloaded from Gene Set Enrichment Analysis (hallmark-hypoxia).

GRHPR SLC2A5 NDRG1 CHST2 DTNA IDS PPP1R3C TGM2 TPBG MT1E ADORA2B CASP6 GBE1 MIF CSRP2 SULT2B1 GAPDHS ETS1 RRAGD B4GALNT2 HAS1 KDELR3 GCK PGM2 CP DDIT3 MXI1 SLC37A4 SAP30 PGAM2 PGK1 AK4 DUSP1 DPYSL4 ANKZF1 STC1 LXN ALDOC ERRFI1 HMOX1 MYH9 PDK3 GALK1 SELENBP1 NEDD4L PFKP NCAN GPI PPP1R15A ATP7A PAM CCN5 ENO1 IGFBP1 SCARB1 F3 PDGFB NDST2 HOXB9 NDST1 SERPINE1 CCN1 SDC2 FBP1 LALBA VHL KLHL24 MAFF ANGPTL4 PYGM SDC4 ACKR3 HS3ST1 PGM1 JUN ALDOA PDK1 NAGK UGP2 IRS2 PPFIA4 PGF PHKG1 IGFBP3 PCK1 PKLR CCN2 KLF6 DCN HDLBP BCL2 LOX FOSL2 NOCT IER3 JMJD6 KIF5A EFNA1 BNIP3L PPARGC1A XPNPEP1 BRS3 SLC2A3 CDKN1B INHA CDKN1C FOXO3 ATF3 CA12 STC2 PLAUR BHLHE40 BCAN SIAH2 B3GALT6 EFNA3 TPI1 KLF7 PLIN2 CDKN1A CXCR4 LARGE1 P4HA2 GPC1 WSB1 EXT1 GYS1 SLC25A1 ZNF292 AKAP12 MAP3K1 DDIT4 TGFBI NR3C1 STBD1 BGN ZFP36 PIM1 TPST2 EGFR AMPD3 PNRC1 ILVBL CCNG2 HK1 FOS TES COL5A1 GPC3 PRDX5 GPC4 ALDOB KDM3A HSPA5 ADM SLC6A6 PKP1 PFKFB3 TPD52 VLDLR NFIL3 TKTL1 TIPARP ISG20 SLC2A1 GAPDH EDN2 CITED2 ENO3 FAM162A BTG1 TGFB3 LDHA ANXA2 RBPJ HK2 PLAC8 TNFAIP3 HEXA PRKCA CAVIN1 CHST3 RORA PFKL GAA GLRX P4HA1 GCNT2 ERO1A SDC3 IL6 CAVIN3 TMEM45A LDHC ENO2 CAV1 SRPX MT2A VEGFA S100A4
